# Gun-Shot Wound through the Heart

**Published:** 1865-07

**Authors:** W. Ellery Bowman


### GUN-SHOT WOUND THROUGH THE HEART.

A THESIS FOR THE DEGREE OF DOCTOR OF MEDICINE, PRESENTED TO THE FACULTY OF RUSH MEDICAL COLLEGE, JANUARY 25TH, 1865.

By W. ELLERY BOWMAN.

Under this somewhat startling heading, I shall devote the following pages to the report of a case which happened to be placed within my observation, and which I watched throughout with the greatest interest; and although in pursuing this course I have perhaps deviated from the ordinary custom, I trust an account of the case will be not unacceptable to the

Faculty of Rush Medical College, and if the paper have any merits, brevity shall, at least, be one of them.

On the 8th of Sept., 1864, about 8 o'clock P. M., in the town of Quincy, Ills., ———, a soldier, aged 28, and apparently healthy, while in a difficulty with a citizen had several wounds inflicted upon him by pistol shots.

My preceptor, who was in charge of one of the Military Hospitals in that place, being summoned, I was allowed to accompany him.

The patient, who had by this time been taken into a house, was evidently laboring under severe shock. The skin was deadly pale, and covered with a profuse cold sweat. The features were contracted and shrunken, and the countenance wore an expression of great anxiety and pain. The pupils were widely dilated. The pulse was absent at the wrist; and on applying the ear over the heart, its action was found to be very feeble and irregular. The respiration was slow and labored, with occasional sighing.

Consciousness was not entirely lost, for the patient complained frequently of an intense burning pain, which he referred to the right lung; and asked to be placed in various positions—that on the left side being most comfortable.

There was intense and unquenchable thirst. Stimulants, as brandy, carbonate of ammonia, etc., were administered, with small quantities of ice water, to allay thirst. Under this treatment the pulse became at times faintly perceptible, but as often disappeared. As soon as arrangements could be made, the man was carried on a litter to the hospital and placed in bed.

On examination, it was found that three balls had taken effect; one passing through the lower border of the anterior wall of the right axillary space, inflicting an insignificant wound; another entering the throax two inches externally to the inferior angle of the right scapula, having perforated the eighth rib; while the third had taken effect upon the left lateral aspect of the abdomen, midway between the last rib and the crest of the ilium, and passed transversely inwards. From the great depression present, and the direction of the wound in the chest, it was feared that the heart had been injured. Vomiting now occurred; at the end of which, a little bright red blood appeared, not mixed with ejecta. Morphia was given in full doses to allay pain.

On account of dyspnœa in the horizontal position, it became necessary to place the patient in a semi-sitting posture, whereby he was enabled to breathe more freely. By the continuous use of stimulants, frictions and warmth, some degree of reaction was obtained. The pulse returned at the wrist and became stronger, though yet exceedingly weak; warmth came back to the surface, and the skin became dryer. These favorable symptoms, however, did not last. The patient vomited everything given him, both food and medicine.

In a few hours the skin again became cold, the pulse more feeble, and prostration again ensued. Dyspnœa was urgent, and blood, in small quantities, was frequently expectorated. The pain in the right side of the chest was severe and burning, and there was crepitation in the right lung, with some dullness on percussion. The thirst remained unabated, and seemed utterly unquenchable by any means that could be used. There had been no external hemorrhage, excepting from the wound in the chest, and from this the bleeding had been inconsiderable. The day and succeeding night wore away without any observable change in the symptoms for better or worse.

The next morning (the 10th) nature made a last, desperate attempt to throw off the load under which she now began to stagger. But the reaction in this instance was even more imperfect than the first, and depression quickly reasserted its sway. The powers of life were now failing perceptibly. Breathing was more difficult, and could only be performed in the sitting posture. The skin assumed a somewhat livid hue, from imperfect æration of the blood, and felt cold and clammy to the touch. There was vomiting of stercoraceous matter, mixed with some blood, which had probably been brought up from the air passages and swallowed. There was a bulging of the lower part of the chest, in front and on the right side, with dullness on percussion over the right lung, and also over an unnatural extent in the præcordia.

The signs of engorgement were present in the left lung, as revealed by auscultation; and the sounds of the heart were so faint as to be heard with difficulty. During this day, sensibility seemed almost completely lost. But little complaint was made of pain, and involuntary and unconscious evacuations from the bowels took place.

At seven o'clock P. M. of Sept. 10th, the patient quietly breathed his last, after having survived the reception of his injuries forty-seven hours.

A post-mortem examination, instituted the next A. M., revealed the following:

A probe passed into the wound, which has been described as situated two inches externally to the inferior angle of the right scapula, took a direction diagonally across the thoracic cavity, from behind forwards and to the left, in a line with the position of the heart. On opening the thorax, both pleuræ were found to contain blood; the right, about f ℥ xvj, and the left a smaller quantity, probably half as much. Both lungs were engorged, and in the track of the bullet through the right lung were numerous spicula of bone from the fractured rib.

The pericardium was moderately distended with blood, portions of which had probably escaped into the pleural cavities through the apertures made by the passing ball. After traversing the right lung, the ball had entered the pericardium, and perforating the left auricle of the heart at its upper and back part, passed across the cavity, pierced the anterior segment of the mitral valve, and reaching the anterior wall of the left ventricle, passed through this at about its middle. Then escaping from the pericardial sac, it could be traced no further. There was no mark of it on the wall of the chest, in the line of its course. It was not in the bottom of the pleural cavity, where it might have fallen after its force was expended. It might have lodged in that part of the left lung which overlaps the heart in front, and the lung collapsing when the chest was laid open, the relations of the parts to each other were so altered that in the haste with which the examination was necessarily conducted this fact was overlooked.

Opposite the wound in the left side of the abdomen an opening was found in the descending colon. As there was no perforation of the opposite side of the intestine, it was evident that the ball had dropped into the bowel; and the whole length of the large intestine was searched for the missile, but without success. It had most likely passed out with the dejections and thus escaped observation.

The extreme depression which the case had exhibited was now readily explained. It was really wonderful that vitality had been sustained so long. The man had lived two days with a bullet wound traversing his heart completely. Judging from the wounds, however, the ball was of small size, and had passed through the organ in a line more nearly parallel with its longitudinal than its transverse diameter, thereby inflicting less injury than it would had it passed transversely. As it was, death seemed to result not so much from the lesion of the heart as from apnœa, occasioned by hemorrhage into the pleuræ and lung. The mode of death was apparently a combination of apnœa and asthenia; the breathing becoming more difficult in proportion as the pleuræ became filled with blood; and the paralyzed and bleeding heart struggling on until fatally embraced by the accumulating "life fluid" (now turned a destroyer) in the cavity of the pericardium.

